# Programmed Death-Ligand 1 Expression in Lung Cancer and Paired Brain Metastases—a Single-Center Study in 190 Patients

**DOI:** 10.1016/j.jtocrr.2022.100413

**Published:** 2022-09-20

**Authors:** Alexandra Kündig, Philipp Zens, Christian Fung, Amina Scherz, Ferdinando Cerciello, Evelyn Herrmann, Ekin Ermis, Ralph A. Schmid, Erik Vassella, Sabina Berezowska

**Affiliations:** a Institute of Pathology, University of Bern, Bern, Switzerland; b Present Address: Department of Otorhinolaryngology-Head and Neck Surgery, Kantonsspital St. Gallen, St. Gallen, Switzerland; c Graduate School for Health Sciences, University of Bern, Bern, Switzerland; d Department of Neurosurgery, Inselspital, Bern University Hospital, University of Bern, Bern, Switzerland; e Present Address: Department of Neurosurgery, Medical Center, University of Freiburg, Freiburg, Germany; f Department of Medical Oncology, Inselspital, Bern University Hospital, University of Bern, Bern, Switzerland; g Department of Radiation Oncology, Inselspital, Bern University Hospital, University of Bern, Bern, Switzerland; h Division of General Thoracic Surgery, Inselspital, Bern University Hospital, University of Bern, Bern, Switzerland; i Department of Laboratory Medicine and Pathology, Institute of Pathology, Lausanne University Hospital and University of Lausanne, Lausanne, Switzerland

**Keywords:** Brain metastasis, Lung cancer, NSCLC, PD-L1, Immune checkpoint inhibitors

## Abstract

**Introduction:**

Expression of programmed death-ligand 1 (PD-L1) is the only routinely used tissue biomarker for predicting response to programmed cell death protein 1/PD-L1 inhibitors. It is to date unclear whether PD-L1 expression is preserved in brain metastases (BMs).

**Methods:**

In this single-center, retrospective study, we evaluated PD-L1 expression using the SP263 assay in consecutively resected BMs of lung carcinomas and paired primary tumors, diagnosed from 2000 to 2015, with correlation to clinicopathological and molecular tumor and patient characteristics.

**Results:**

PD-L1 tumor proportional score (TPS) could be evaluated on whole tissue slides in 191 BMs and 84 paired primary lung carcinomas. PD-L1 TPS was less than 1% in 113 of 191 (59.2%), 1% to 49% in 34 of 191 (17.8%), and greater than or equal to 50% in 44 of 191 (23.0%) BMs. TPS was concordant between BMs and paired primary lung carcinomas in most cases, with discordance regarding the clinically relevant cutoffs at 1% and 50% in 18 of 84 patients (21.4%). Four of 18 discordant cases had no shared mutations between the primary lung carcinoma and BM. Intratumoral heterogeneity, as assessed using tissue microarray cores, was only significant at the primary site (*p*_Wilcoxon signed rank_ = 0.002) with higher PD-L1 TPS at the infiltration front (mean = 40.4%, interquartile range: 0%–90%). Neither TPS greater than or equal to 1% nor TPS greater than or equal to 50% nor discordance between the primary lung carcinoma and BMs had prognostic significance regarding overall survival or BM-specific overall survival.

**Conclusions:**

PD-L1 expression was mostly concordant between primary lung carcinoma and its BM and between resections of BM and stereotactic biopsies, mirrored by tissue microarray cores. Differences in PD-L1 TPS existed primarily in cases with TPS greater than 10%, for which also human assessment tends to be most error prone.

## Introduction

Lung cancer is the deadliest malignant disease worldwide and the second most often diagnosed cancer.^1^ It often presents in advanced stages with a 5-year survival rate of 10% to 20% in most countries.^1^ Lung cancer is the most frequent origin of brain metastases (BMs). Up to 40% of patients with non-small cell lung carcinoma (NSCLC) develop BMs during the course of their disease.^2^ Although small cell lung carcinoma (SCLC) is most susceptible to metastasize to the brain, most BMs originate from adenocarcinoma, owing to its overall frequency.^3^

Therapy of NSCLC includes a combination of surgery, chemotherapy, and, where applicable, radiotherapy, depending on stage, histologic tumor type, and patient characteristics.^4^ Likewise, the treatment of lung cancer BMs combines both local (surgery, radiotherapy) and systemic approaches.^5^ Surgical intervention is generally reserved for cases with a single metastasis, symptomatic mass effects, or diagnostic uncertainty.^5^ Whereas whole-brain radiotherapy (WBRT) has been the standard of care for many decades, stereotactic radiosurgery (SRS) has now replaced it in many clinical settings.^5^ WBRT is considered for patients in whom SRS is not recommended.^5^ When using WBRT, strategies such as hippocampus avoidance with Memantine can be offered to reduce possible neurocognitive decline.^5^ In the past decade, SRS alone has become the standard of care for patients with good performance and one to four newly diagnosed BMs.^6^ Data are evolving for patients with 5 to 20 metastases.^6^ The identification of molecular drivers in NSCLC has resulted in systemic targeted therapeutic approaches, and the availability of several novel agents with better brain penetration has enabled its usage in the treatment of BM. According to current guidelines, in patients with asymptomatic BM and targetable alterations such as *EGFR*, *ALK*, or *ROS1*, systemic targeted treatments should be considered upfront.^5^

In recent years, immune checkpoint inhibitors (ICIs) have become an important additional cornerstone in the treatment of advanced NSCLC.^4^ Programmed cell death protein 1, found on the surface of T-cells and B-cells, mediates inhibitory signals.^7^ Its ligand, programmed death-ligand 1 (PD-L1), is expressed by a multitude of cancers leading to immune evasion.^7^ Recently, pembrolizumab was found to be active in BMs from NSCLC with PD-L1 expression in the primary tumor, similar to its systemic activity.^8^ In this cohort of 27 patients with PD-L1 expression of at least 1% in the primary NSCLC, in whom systemic and central nervous system responses could be evaluated, six patients were found to have discordant responses in BM versus the primary lung tumor burden.^8^ PD-L1 status in BMs was not reported.^8^

PD-L1 expression is the only tissue biomarker used in clinical routine for selecting patients for ICI therapy. Nevertheless, current literature is ambivalent about its preservation in BM, as summarized in Table 1.^9^, ^10^, ^11^, ^12^, ^13^, ^14^, ^15^, ^16^ Some studies report discordant expression to be associated with a longer time span after which the BM occurred,^9^^,^^12^ but most studies lack clinical data including information on therapeutic interventions (e.g., radiotherapy or chemotherapy) between tissue sampling.^9^^,^^12^^,^^15^^,^^16^ Furthermore, most studies investigated discordance in PD-L1 expression using a 5% cutoff and lack information on the cutoffs 1% and 50% currently used for clinical decision-making.^4^ In addition, different PD-L1 antibody clones were applied, which might be one reason for the discordant results.^9^, ^10^, ^11^, ^12^, ^13^, ^14^, ^15^^,^^17^

**Table 1:** Literature Review Summarizing the Results of Current Studies Assessing PD-L1 Expression (TPS) in Paired Samples of Primary Lung Carcinomas and Brain Metastases

Study	Year	Number of Patients With Paired Samples (Lung Tumor and Brain Metastasis)	Histologic Tumor Type	Treatment	PD-L1 Clone	Tissue	PD-L1 Expression (TPS)
Mansfield^9^	2016	73	NSCLC/SCLC	n.a.	E1L3N	Resection and biopsy samples Whole tissue slides	14% discordance (5% cutoff)
Berghoff^10^	2016	4	SCLC	n.a.	5H1	Resection samples Whole tissue slides	25% discordance (1% cutoff)
Takamori^11^	2017	16	NSCLC	Conversion to PD-L1 positivity after radiotherapy	SP142	Resection and biopsy samples Whole tissue slides	24% discordance (5% cutoff)
Zhou^12^	2018	25	NSCLC	n.a.	6E8	Resection and biopsy samples Whole tissue slides	28% discordance (5% cutoff)
Kim R^13^	2019	12	NSCLC/SCLC	No impact	E1L3N	Resection and biopsy samples Whole tissue slides	High concordance of H-scores, no further details
Téglási^14^	2019	61	LUAD	No impact	SP142	Tissue microarray	High concordance, no exact numbers on percentage of discordant cases
Batur^15^	2020	24	NSCLC	No therapy in between tissue acquisition	22C3	Resection and biopsy samples Whole tissue slides	High concordance 20.8% discordance (1% cutoff)
Song^16^	2021	28	NSCLC/SCLC	No previous therapy	SP263	Resection and biopsy samples Whole tissue slides	High concordance 14.28 discordance (TPS > 0 vs. TPS = 0)

LUAD, lung adenocarcinoma; n.a., not applicable; PD-L1, programmed death-ligand 1; TPS, tumor proportional score.

Our aim was therefore to evaluate PD-L1 expression in paired primary lung carcinomas and BMs and to set these results into context with clinical data. Because sometimes the only tissue available for biomarker analysis might derive from a BM, a strong concordance between primary and metastatic tumors would indicate that a sample from one site is representative of the immunobiological status of the other site.

## Materials and Methods

### Patient Cohort

Initially, the study cohort comprised 212 patients with consecutively resected lung carcinoma BMs, diagnosed at the Institute of Pathology, University of Bern, during 2000 to 2015.^40^ All BMs were resected at the Department of Neurosurgery, Inselspital University Hospital Bern. A total of 191 patients was finally included for analysis after excluding two cases owing to origin other than lung cancer as re-evaluated according to the clinical files, 18 cases owing to insufficient available tissue (i.e., <100 tumor cells^18^), and one case where tissue was available only from the spinal cord.^39^, ^40^ In 14 of 191 cases (7.3%), more than one BM was used for evaluation of PD-L1 expression, and in another three cases, more than one slide was available for analysis. In 84 of 191 cases (44.0%), paired tissue from the primary tumor was available for PD-L1 analysis. A flowchart summarizing the patient cohort and tissue included for analysis is provided as Supplementary Figure 1. The cohort was initially assembled according to the pathologic reports and validated using clinical files, information from the cancer registries, and the patients’ general practitioners.^39^ The histologic tumor type was re-evaluated in all cases according to the current guidelines.^19^ All primary lung carcinomas with sufficient information in the pathologic reports were restaged according to the Union for International Cancer Control eighth edition of the TNM classification.^20^ Furthermore, information about the initial clinical stage was included wherever available. The initial clinical stage was available for 82 of 191 patients (42.9%) and the pathologic T classification for 84 of 191 patients (44.0%). Patients with clinical stages I to III at initial diagnosis were considered as having early stage tumors. SCLC cases with extensive disease at initial diagnosis were included as stage IV cases.

Synchronicity was defined as the clinical diagnosis of lung primary tumor and BM within 3 months.^21^ BMs were synchronous in 70 of 191 patients (36.6%) and metachronous in 77 of 191 patients (40.3%). Information on the date of the primary tumor was lacking for 44 of 191 patients (23.0%). Smoking status at diagnosis was available for 190 of 191 patients (99.5), and 113 patients were active smokers. Table 2 summarizes the detailed cohort characteristics according to PD-L1 expression.

**Table 2:** Baseline Characteristics of the Study Cohort According to PD-L1 Status in BMs, as Evaluated in Whole Tissue Sections

Characteristics	PD-L1 < 1% (n = 113)	PD-L1 1%–49% (n = 34)	PD-L1 ≥ 50% (n = 44)	*p* Value
Age, y, median (range)	60 (30–80**)**	62 (40–82**)**	60.5 (40–80**)**	0.923^a^
Sex	n = 113 (%)	n = 34 (%)	n = 44 (%)	0.443^b^
Female	39 (34.5)	12 (35.3)	20 (45.5)	
Male	74 (65.5)	22 (64.7)	24 (54.5)	
Smoking status	n = 112 (%)	n = 34 (%)	n = 44 (%)	0.864^b^
Never	9 (8)	1 (2.9)	4 (9.1)	
Former	38 (33.9)	12 (35.3)	13 (29.5)	
Active	65 (58.1)	21 (61.8)	27 (61.4)	
Pack-years, median (range)	40 (1–120)	40 (10–100)	40 (3–100)	0.451^a^
Histologic Tumor Type	n = 113 (%)	n = 34 (%)	n = 44 (%)	<0.001^b^
LUAD	64 (56.6)	28 (82.6)	40 (90.9)	
LUSC	14 (12.4)	5 (14.7)	4 (9.1)	
LCC	2 (1.8)	1 (2.9)		
SCLC	20 (17.7)			
LCNEC	7 (6.2)			
Other	6 (5.3)			
Systemic therapy affecting PD-L1 assessment	n = 100 (%)	n = 31 (%)	n = 42 (%)	0.498^b^
None	53 (53)	20 (64.5)	27 (64.3)	
Ctx before samples	28 (28)	6 (19.4)	5 (11.9)	
TKI before samples			1 (2.4)	
Ctx/TKI before samples	1 (1)	1 (3.2)		
Ctx between P/BM	13 (13)	4 (12.9)	8 (19.1)	
Ctx/TKI between P/BM	1 (1)			
Ctx between BM/P	2 (2)			
Ctx between BM	2 (2)		1 (2.4)	
Radiotherapy affecting PD-L1 assessment	n = 110 (%)	n = 33 (%)	n = 44 (%)	0.669
None	72 (65.5)	24 (72.7)	37 (84.1)	
Thoracic before samples	8 (7.3)	1 (3)	1 (2.3)	
Cranial before samples	7 (6.4)	2 (6.1)		
Thoracic/cranial before samples	5 (4.5)		2 (4.5)	
Thoracic between P/BM	5 (4.5)	1 (3)	1 (2.3)	
Cranial between P/BM	1 (0.9)			
Cranial between BM/P	5 (4.5)	4 (12.1)	1 (2.3)	
Cranial between BM	5 (4.5)	1 (3)	1 (2.3)	
Other	2 (1.8)		1 (2.3)	
pT descriptor	n = 49 (%)	n = 16 (%)	n = 19 (%)	0.471^a^
pT0	1 (2)	1 (6.3)		
pT1	8 (16.3)	6 (37.5)	4 (21.1)	
pT2	23 (46.9)	3 (18.7)	7 (36.8)	
pT3	7 (14.3)	4 (25)	4 (21.1)	
pT4	10 (20.4)	2 (12.5)	4 (21.1)	
pN descriptor	n = 50 (%)	n = 17 (%)	n = 19 (%)	0.654^a^
pN0	18 (36)	6 (35.3)	4 (21.1)	
pN1	15 (30)	7 (41.2)	9 (47.4)	
pN2	16 (32)	4 (23.5)	5 (26.3)	
pN3	1 (2)		1 (5.3)	
cStage	n = 48 (%)	n = 16 (%)	n = 18 (%)	0.06^a^
Stage I	4 (8.3)	1 (6.3)		
Stage II	6 (12.5)	2 (12.5)		
Stage III	6 (12.5)	2 (12.5)	1 (5.6)	
Stage IV	32 (66.7)	11 (68.7)	17 (94.4)	
Number of BM (median [range])	1 [1–14]	1 [1–14]	1 [1–9]	0.908^a^
Latency to BM	n = 86 (%)	n = 27 (%)	n = 34 (%)	0.212^b^
Synchronous	36 (41.9)	14 (51.9)	20 (58.8)	
Metachronous	50 (58.1)	13 (48.1)	14 (41.2)	
Location BM	n = 113 (%)	n = 34 (%)	n = 43 (%)	0.406^b^
Frontal	36 (31.9)	12 (35.3)	10 (23.3)	
Temporal	9 (8)	3 (8.8)	3 (7)	
Parietal	6 (5.3)	5 (14.7)	5 (11.6)	
Occipital	9 (8)	5 (14.7)	5 (11.6)	
Cerebellum	42 (37.2)	6 (17.6)	12 (27.9)	
Frontotemporal	1 (0.9)			
Frontoparietal	2 (1.8)	1 (2.9)	1 (2.3)	
Temporoparietal	1 (0.9)			
Temporo-occipital	1 (0.9)		1 (2.3)	
Parieto-occipital	4 (3.5)		2 (4.7)	
Meninges			1 (2.3)	
Other	2 (1.8)	2 (5.9)	3 (7)	
Molecular subgroup	n = 27 (%)	n = 11 (%)	n = 15 (%)	0.265^b^
P = BM	6 (22.2)	2 (18.2)	2 (13.3)	
P > BM	4 (14.8)	2 (18.2)	6 (40)	
P < BM	8 (29.6)	4 (36.4)	1 (6.7)	
P ∩ BM	8 (29.6)	3 (27.3)	3 (20)	
P / BM	1 (3.7)		3 (20)	

BM, brain metastasis; cStage; clinical stage; Ctx, chemotherapy; LCC, large cell carcinoma; LCNEC, large cell neuroendocrine carcinoma; LUAD, lung adenocarcinoma; LUSC, lung squamous cell carcinoma; P / BM, no shared mutations; P < BM, private mutations in the brain metastasis; P = BM, patients with same mutations at the primary and metastatic site; P > BM, private mutations at the primary tumor site; P ∩ BM, intersecting cases with private mutations at both sites; P, primary tumor in the lung; PD-L1, programmed death-ligand 1; pN, pathologic N; pT, pathologic T; TKI, tyrosine kinase inhibitor.

a Kruskal-Wallis ranked sum test.

b Fisher’s test (simulated with 2000 replicates).

Overall survival (OS) was defined as the time from initial diagnosis of the disease to death from any cause. BM-specific OS (BMOS) was defined as the time from diagnosis of the first BM to death of any cause.

The study was conducted and is reported according to the REMARK criteria,^22^ and was approved by the Cantonal Ethics Commission of the Canton of Bern, which waived the requirement for written informed consent (KEK-BE: 2016-01497).

### Tissue Microarray

When sufficient material was available, a tissue microarray (TMA) was constructed from FFPE tissue blocks as described elsewhere, using an automated tissue microarrayer (Grandmaster, 3DHistech, Budapest, Hungary).^23^^,^^24^ In short, after review of the hematoxylin and eosin-stained slides, digitization, and annotation, eight tissue cores (diameter = 0.6 mm) from the tumor center and whenever possible four cores from the infiltration front were transferred to the TMA acceptor block. The cores from the same tumor were placed on four different TMA acceptor blocks to exclude technical staining bias.

### Immunohistochemistry

For immunohistochemical staining, tissue blocks were cut at 5 μm. PD-L1 staining was performed in a closed system using the Ventana SP263 assay (Roche Diagnostics International AG, Rotkreuz, Switzerland) on the fully automated immunostainer BenchMark ULTRA (Roche Diagnostics International AG) following the manufacturer’s instructions. The sections were preprocessed using CC1 buffer at 100°C for 64 minutes, followed by antibody incubation at 37°C for 16 minutes and visualization with 3,3'-Diaminobenzidine.

The whole slides corresponding to the donor blocks for TMA construction were used for evaluation of PD-L1 expression on whole slides. All selected slides harbored at least 100 tumor cells.^18^ PD-L1 was evaluated applying the tumor proportional score (TPS) used in daily clinical routine, defined as the proportion of positive tumor cells, by an experienced senior pathologist specialized in lung pathology and PD-L1 evaluation (S.B.), in a blinded fashion. TPS was assessed in 1% steps up to 10% and in 5% steps for cases greater than 10%. Furthermore, TPS was threefold categorized in the clinically significant bins of less than 1%, 1% to 49%, and greater than or equal to 50%. The age of tissue paraffin blocs had no effect on PD-L1 evaluation (*p*_Kruskal-Wallis_ = 0.744; Supplementary Fig. 2).

### Next-Generation Sequencing

Genomic DNA and RNA were extracted from tissue punches using the QIAamp DNA Microkit (Qiagen, Hilden, Germany) and Ambion RevoverAll Kit (ThermoFisher Scientific, Waltham, MA), respectively. Next-generation sequencing was conducted using the Oncomine Comprehensive Panel v3 (ThermoFisher Scientific) and analyzed as reported previously.^25^ The analysis was successful in 54 adenocarcinoma tumor pairs and failed in two primary tumors and one BM. These patients were further categorized into evolutionary subgroups differentiating between cases with all shared mutations (P = BM), private mutations present only in the primary lung carcinoma (P > BM), private mutations available only in the BM (P < BM), mutations present in both the primary lung carcinoma and BM (P ∩ BM), and no shared mutations (P / BM), as previously reported in detail.^25^ The major driver alterations in lung carcinoma and genomic alterations important for evaluating the benefit from ICI are summarized in Supplementary Table 1. All genomic alterations with oncogenic or likely oncogenic effect according to OncoKB (accessed on August 3, 2022) were included.^26^ For detailed description of all genomic alterations observed in these samples, we refer to the Supplementary Material 1 of the molecular study on this cohort.^25^

### Statistical Analysis

R software (version 4.1.3, https://cran.r-project.org) was used for statistical analysis. For comparison of the baseline characteristics, Fisher’s exact test was used for categorical or binary variables and the Kruskal-Wallis test for ordinal or continuous variables. We used the Wilcoxon signed rank test and Spearman correlation for evaluating intercore (only infiltration front) and interregion heterogeneity and for comparing PD-L1 TPS as assessed on whole slides versus TMA and in the primary lung carcinoma versus BMs. For intercore heterogeneity in the tumor center, a random sample of four cores was picked per patient and we used the Friedman test. For visual comparison of no more than two groups, we, in addition, generated Bland-Altman plots.

Discordance between PD-L1 TPS in BMs and primary lung cancers was defined as discordance regarding the 1% or 50% cutoffs, as assessed on whole slides. Wilcoxon ranked sum and Fisher’s exact test were used for testing the association of clinicopathologic parameters with TPS discordance, PD-L1 TPS greater than or equal to 1%, or PD-L1 TPS greater than or equal to 50%.

For survival analyses, survival curves were represented as Kaplan-Meier plots, and the log-rank test was used to evaluate the prognostic potential of PD-L1 TPS in the BM regarding the 1% and 50% cutoff and PD-L1 TPS discordance. Two-sided *p* values of less than 0.05 were regarded as significant.

## Results

### Discordant PD-L1 Expression Between Primary Lung Carcinoma and Its BM on Whole Slides in Less Than 20% of Patients

**Figure 1:**
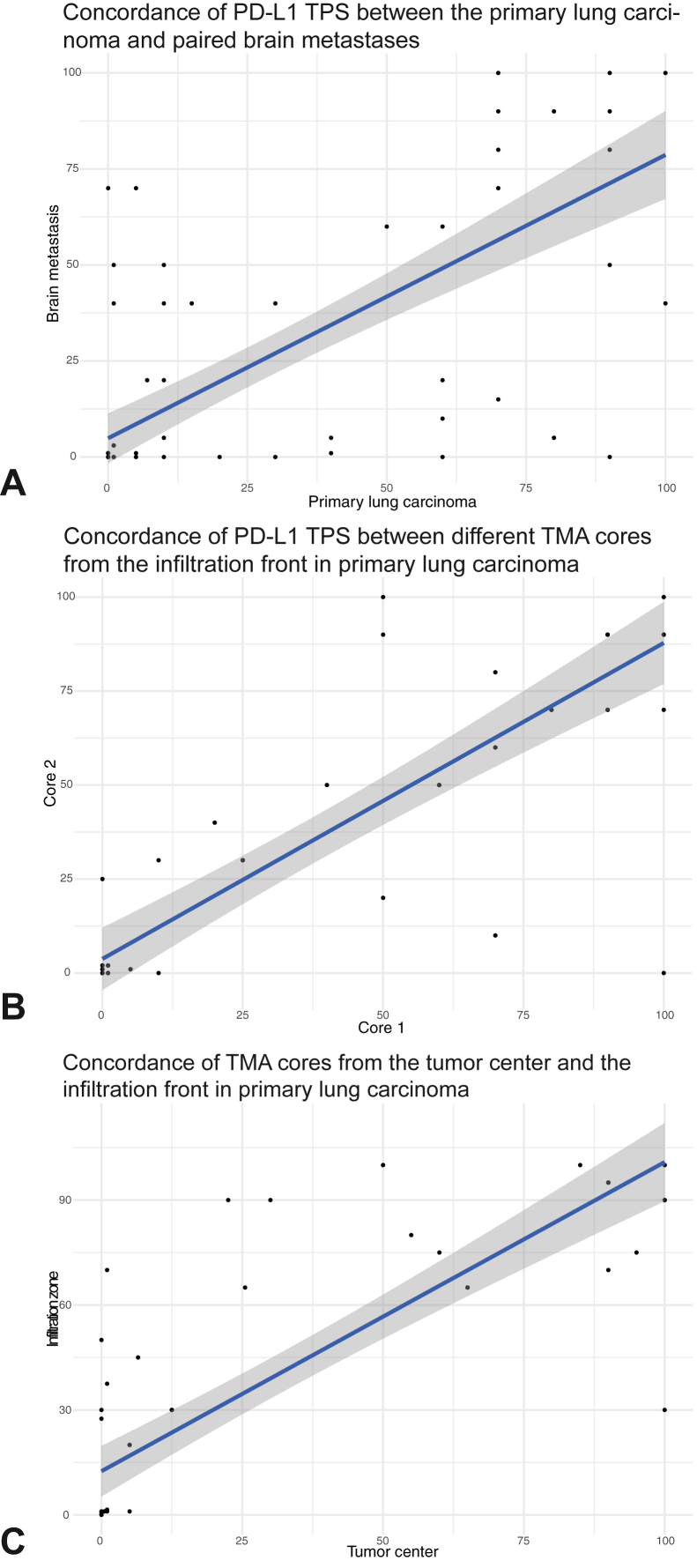
(*A*) Concordance of PD-L1 TPS between the primary lung carcinoma and its brain metastasis, as assessed on whole tissue sections. (*B*) Concordance of PD-L1 TPS between different TMA cores from the infiltration front in primary lung carcinoma. (*C*) Concordance of TMA cores from the tumor center and the infiltration front in primary lung carcinoma. The blue line represents a fitted linear model using PD-L1. PD-L1, programmed death-ligand 1; TMA, tissue microarray; TPS, tumor proportional score.

**Figure 2:**
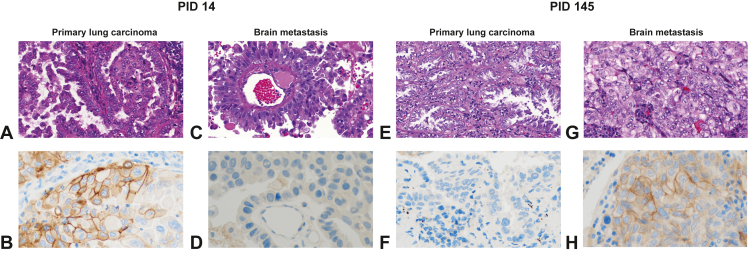
Representative images from two cases with highly discrepant PD-L1 staining between brain metastasis and paired primary lung carcinoma. Case PID14 features TPS 60% in the primary lung carcinoma (*B*) and TPS less than 1% in the brain metastasis (*D*). Case PID145 features TPS less than 1% in the primary lung carcinoma (*F*) and TPS 70% in the brain metastasis (*H*). *A*, *C*, *E*, *G*: H&E; *B*, *D*, *F*, *H*: PD-L1 SP263 CE-IVD. All images at ×40. H&E, hematoxylin and eosin; PD-L1, programmed death-ligand 1; TPS, tumor proportional score.

In the BM specimens, PD-L1 TPS as assessed on whole slides was less than 1% in 113 of 191 patients (59.2%), 1% to 49% in 34 of 191 patients (17.8%), and greater than or equal to 50% in 44 of 191 patients (23.0%). In the paired primary specimens, PD-L1 TPS was less than 1% in 37 of 84 patients (44.0%), 1% to 49% in 21 of 84 patients (25.0%), and greater than or equal to 50% in 26 of 84 patients (31.0%). PD-L1 TPS was generally concordant between lung carcinoma and BM, both when assessing it as a continuous parameter (*p*_Wilcoxon signed rank_ = 0.567, *p*_Spearman_ < 0.001, *r*_Spearman_ = 0.81; Fig. 1*A* and Supplementary Fig. 3) or as a categorized marker (*p* = 0.092). Importantly, 35 of 84 cases (41.7%) were negative (PD-L1 TPS < 1%) both in the primary and metastatic localization. Regarding the clinically relevant cutoffs at 1% and 50%, 18 of 84 patients (21.4%) had a discordant PD-L1 expression between primary lung carcinoma and the BM (Table 3). Among those, three patients (PID14, 111, 145) had discrepancy regarding both cutoffs (1% and 50%) (Fig. 2A-H). Four patients had nonoverlapping molecular profiles according to the next-generation sequencing panel. Excluding these cases with nonoverlapping molecular profiles, 14 of 84 cases (16.7%) with shared molecular background were discordant.

**Table 3:** Discordant Cases Regarding the 1% or 50% Cutoff for PD-L1 Expression

PID	Histologic Subtype	TPS Primary Lung Carcinoma [%]	TPS BM [%]	Latency	Therapy Between Acquisition of Samples	Molecular Subtype
4	LUAD	60	10	Metachronous	Chemotherapy	P < BM
10	LUSC	5	0	Synchronous		
14	LUAD	60	0	Metachronous	Chemotherapy	P = BM
24	LUAD	1	0	Synchronous		
67	LUAD	10	50	Synchronous		
69	LCNEC	1	0	Metachronous	Chemotherapy	
87	LUAD	5	70	Metachronous	Chemotherapy	P / BM
99	LUAD	80	5	Synchronous		P > BM
111	LUAD	90	0	Synchronous		P / BM
115	LUAD	70	15	Synchronous		P ∩ BM
121	LUAD	10	0	Metachronous		P ∩ BM
131	Other	20	0	Metachronous		P ∩ BM
145	LUAD	0	70	Synchronous		P / BM
162	Other	30	0	Synchronous		P = BM
167	LUAD	60	20	Metachronous		
178	LUAD	100	40	Synchronous		
180	LUAD	0	1	Synchronous		P < BM
204	LUAD	1	50	Synchronous		P / BM

Note: Cases for which PD-L1 TPS assessment on whole slides was discordant when considering the clinically relevant 1% and 50% cutoffs. For each cutoff, nine cases were discordant but PID14, PID111 and PID145 were discordant regarding both the 1% and 50% cutoffs. Shaded patients showed a change from <1% PD-L1 expression to ≥50%.

BM, brain metastasis; LCNEC, large cell neuroendocrine carcinoma; LUAD, lung adenocarcinoma; LUSC, lung squamous cell carcinoma; P / BM, no shared alterations; P < BM, additional private alterations in the brain metastasis; P = BM, all alterations shared; P > BM, additional private alterations in the primary lung carcinoma; P ∩ BM, private alterations in the primary lung carcinoma and brain metastases; PD-L1, programmed death-ligand 1; PID, patients’ identification; TPS, tumor proportional score.

In 14 of 191 patients (7.3%), PD-L1 TPS was assessed in two or three BM and in three of 191 patients (1.6%) in more than one slide per BM (Supplementary Fig. 1). Regarding the clinically meaningful cutoffs (1% and 50%), only three patients had discordance: PID23 and PID177 changed from 0% to 1% TPS and PID93 changed from 40% to 50% (different slide but same BM; Supplementary Table 2).

### Patients With No Shared Genetic Alterations Between the Primary Lung Carcinoma and Brain Metastasis Were Discordant Regarding the PD-L1 50% Cutoff

We investigated whether clinicopathologic factors were associated with discordant PD-L1 expression between primary lung carcinoma and the BM regarding the clinically relevant cutoffs 1% and 50% PD-L1 TPS, as assessed on whole slides. We investigated the following parameters: age, sex, smoking status, pack-years, histologic tumor type, clinical stage at initial diagnosis, latency of diagnosis, intervening systemic therapy or cranial radiation with possible effect (WBRT or partial cranial radiation before assessment of the BM), molecular subgroup, and the location of the BM (frontal versus cerebellar location). Higher age was associated with discordant PD-L1 TPS regarding the 1% cutoff (*p*_Wilcoxon ranked sum_ = 0.023; Supplementary Fig. 4), and patients with no shared genetic alterations between the primary lung carcinoma and the BM were all discordant regarding the 50% cutoff (*p*_Fisher’s exact_ < 0.001; Supplementary Table 3). In addition, patients harboring KRAS alterations were more likely to be discordant regarding the 50% cutoff (*p*_Fisher’s exact_ = 0.003).

Furthermore, we assessed the association of these parameters with PD-L1 positivity greater than or equal to 1% or greater than or equal to 50% in the BM. There was no association of the histologic tumor type with PD-L1 TPS, when considering only adenocarcinoma and squamous cell carcinoma, and excluding neuroendocrine tumors, which are known to be PD-L1 negative. Patients with higher initial clinical stage tended to have PD-L1 TPS greater than or equal to 50% (*p*_Wilcoxon ranked sum_ = 0.019) as did patients with no shared mutations between the primary lung carcinoma and BM (*p*_Fisher’s exact_ = 0.037).

### Higher Discordance of PD-L1 Expression Between TMA Cores and Whole Slides in the Primary Lung Carcinoma Site Than in BM

PD-L1 expression in TMA cores, which model stereotactic biopsies, was compared with whole slide evaluation. For 187 of 191 patients (97.9%) with whole slide assessment of PD-L1 TPS in the BM, at least one TMA core could be evaluated, resulting in a total of 1599 cores (median of eight cores per tumor, range: 1–12). In 72 of 84 patients (85.7%) with paired assessment of PD-L1 expression on the whole slide, at least one TMA core could be evaluated for the primary lung carcinoma, resulting in a total of 713 cores (median of 10, range: 5–12). These cases were used to test for concordance between PD-L1 TPS evaluated using the TMA cores compared with whole tissue sections.

PD-L1 expression between the TMA cores and whole tissue slides was compared using the median over all assessed TMA cores per patient and site. There was no statistical discordance when comparing PD-L1 TPS in BM (*p*_Wilcoxon signed rank_ = 0.05, *p*_Spearman_ < 0.001, *r*_Spearman_ = 0.91; Supplementary Fig. 5*A* and *B*). Nevertheless, in the primary lung carcinoma, TPS scores differed significantly when comparing assessment on TMA cores with whole tissue slides (*p*_Wilcoxon signed rank_ = 0.001, n = 75, *p*_Spearman_ < 0.001, *r*_Spearman_ = 0.94; Fig. 1*B* and Supplementary Fig. 5*C* and *D*) using the Wilcoxon signed rank, but they were highly correlated when using Spearman correlation.

### Higher Heterogeneity of PD-L1 Expression Within Primary Lung Carcinoma Than BM

We tested for heterogeneity between individual TMA cores (within the same tumor region) and for heterogeneity between tumor center and infiltration front separately in BM and primary lung carcinoma. Because a different number of cores covering the tumor center was evaluated in some patients (missing core, no tumor tissue present in the core, etc.), four random cores from the tumor center and two random cores from the infiltration front were selected for each case to test for heterogeneity using the Friedman test. For testing interregion heterogeneity, all patients with available cores, even when below the threshold for intercore testing, were included.

In the group of BMs, seven patients had less than four cores covering the tumor center of the BM. Of the 102 patients with assessed TMA cores at the infiltration front, 12 patients were excluded from heterogeneity testing in the infiltration front because only one core had been evaluated. There was no intercore heterogeneity, neither between cores from the tumor center (*p* = 0.606; Supplementary Fig. 6*A*) nor between cores from the infiltration front (*p*_Wilcoxon signed rank_ = 0.451, *p*_Spearman_ < 0.001, *r*_Spearman_ = 0.88; Supplementary Fig. 6*B*), and no interregion heterogeneity (*p* = 0.965, *p*_Spearman_ < 0.001, *r*_Spearman_ = 0.93; Supplementary Fig. 6*C*).

In the group of primary lung carcinomas, all patients had enough cores evaluated from the tumor center but 13 of 60 patients were excluded owing to only one core being available from the infiltration front. There was no intercore heterogeneity neither between cores from the tumor center (*p* = 0.769; Supplementary Fig. 7*A*) nor between cores from the infiltration front (*p*_Wilcoxon signed rank_ = 0.678, *p*_Spearman_ < 0.001, r_Spearman_ = 0.95; Fig. 1*B*). Nevertheless, there was interregion heterogeneity in primary lung carcinoma (*p*_Wilcoxon signed rank_ = 0.002, *p*_Spearman_ < 0.001, *r*_Spearman_ = 0.9; Fig. 1*C* and Supplementary Fig. 7*B*).

### No Prognostic Significance of PD-L1 Expression in BM

**Figure 3:**
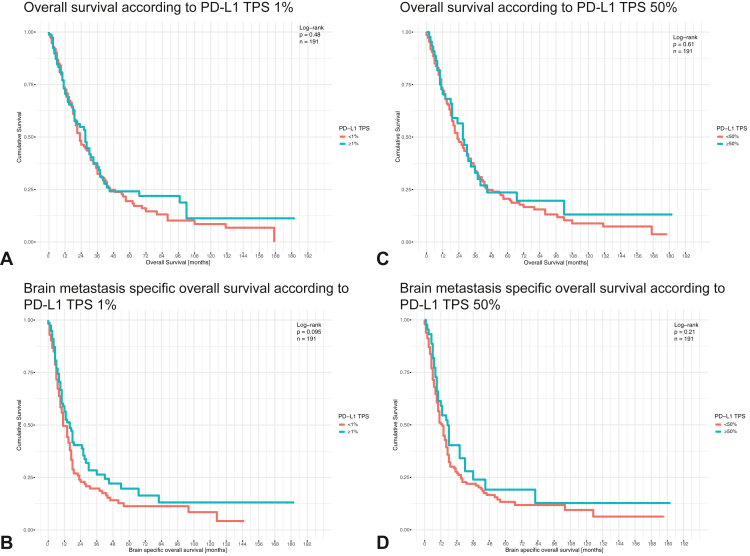
Kaplan-Meier plots depicting the overall survival (*A*, *C*) and brain-specific overall survival (*B*, *D*) according to the PD-L1 TPS cutoffs 1% (*A*, *B*) and 50% (*C*, *D*). PD-L1, programmed death-ligand 1; TPS, tumor proportional score.

All 191 patients with available PD-L1 TPS in the BM were included in the survival analysis. Median OS from time of initial diagnosis was 24 months (95% confidence interval: 20–30, 157 events). Median BMOS from time of resection of the BM was 14 months (95% confidence interval: 11–16, 157 events). PD-L1 expression assessed in BM had no prognostic significance, neither for OS (*p*_1%_ = 0.48, *p*_50%_ = 0.61) nor for BMOS (*p*_1%_ = 0.095, *p*_50%_ = 0.21), neither regarding the 1% (Fig. 3*A* and *B*) nor the 50% (Fig. 3*C* and *D*) cutoff. Cases with discordant PD-L1 TPS had no difference in OS (*p* = 0.37; Supplementary Fig. 8*A*) nor BMOS (*p* = 0.15; Supplementary Fig. 8*B*).

## Discussion

PD-L1 is to date the only biomarker used for selecting patients for ICI.^4^ After excluding patients with untreated or progressing lung cancer BM from ICI clinical trials, it was only recently that pembrolizumab was found to be effective in this patient group, with response rates in the brain similar to systemic response.^8^ Nevertheless, some patients had discrepant responses between the brain and the lung, raising the question of concordance of PD-L1 expression in the different tumor localizations, which we addressed in our current study.

In our cohort of 84 consecutive primary lung carcinomas with paired BM, PD-L1 expression was overall concordant in BM and lung carcinomas, when evaluating TPS using the clinically significant cutoffs 1% and 50%.^8^^,^^27^ Though discordance was statistically not significant, it was present in 14 patients (16.7%), excluding four patients without any shared genomic alteration between the primary carcinoma and BM. Discordance was neither associated with the timing of metastasis diagnosis (synchronous versus metachronous) nor with initial clinical stage.

PD-L1 expression in paired samples of BM and primary lung cancer has been previously investigated with incongruent results, as summarized in Table 1.^9^, ^10^, ^11^, ^12^, ^13^, ^14^, ^15^, ^16^ Our study reports the largest sample size in a very well characterized cohort including full clinicopathologic reevaluation according to the current guidelines. Another strength of our approach is the usage of a standardized and CE-IVD marked PD-L1 assay and the application of the routinely used TPS readout including the clinically significant cutoffs 1% and 50%.

In two studies using the TPS cutoff 5% and laboratory-developed tests, one explanation for discordance was a longer period between tissue acquisition.^9^^,^^12^ This had no impact in our cohort. The different PD-L1 expression cutoff, the usage of different PD-L1 antibody clones, and sample size might explain the differing results.^28^^,^^29^

Téglási et al.^14^ observed a strong positive PD-L1 correlation between 61 paired samples regarding cutoff levels 1%, 5%, and 50%, unaffected by any antecedent therapies, in line with our results. Nevertheless, the SP142 antibody used in this study is the only one explicitly not recommended for PD-L1 TPS assessment in lung cancers.^17^

In discrepant cases in our cohort, BMs were more often PD-L1 negative. The unique immunosuppressive microenvironment of the brain might contribute to this observation.^30^ It was found that interferon-γ (IFN-γ) can up-regulate PD-L1 expression, and it remains unclear whether PD-L1 expression would be more frequent if more lymphocytes could reach the metastatic site.^9^^,^^31^ Takamori et al.^11^ found that radiotherapy may contribute to a positive conversion in metastases of PD-L1–negative NSCLC, but their cohort was small, with only two of 16 patients having this effect. In our cohort, five of 18 discrepant cases featured a higher PD-L1 expression in the BM, which was not explicable by previous radiotherapy.

PD-L1 expression correlates with driver mutations in NSCLC.^32^ KRAS mutation may induce PD-L1 expression through phospho-ERK signaling, making ICI an interesting therapeutic strategy in KRAS-mutant adenocarcinomas.^32^ In our cohort, patients harboring KRAS alterations were more likely to be discrepant only regarding the 50% cutoff. Contrary to Falk et al.,^33^ who described an association of PD-L1 TPS greater than or equal to 1% with KRAS mutations, KRAS mutations had no association with PD-L1 TPS in our cohort. Variants of KRAS mutations were found to lack association with higher PD-L1 TPS.^34^

In advanced NSCLC, small biopsies rather than resections are available for diagnosis and biomarker assessment. As PD-L1 is known to be heterogeneously expressed in NSCLC, it remains disputable whether biopsy samples are suitable for assessing PD-L1 expression.^29^^,^^35^ Our data point toward a higher heterogeneity of PD-L1 expression in primary lung carcinomas compared with BM. Discrepancies of PD-L1 TPS scores were noted between infiltration front and tumor center in the primary lung tumors. When comparing TMA cores and whole slides, TPS scores differed significantly in the primary tumor only. Nevertheless, all these assessments, comparisons of different TMA cores of the infiltration front, different regions (tumor center versus infiltration front), and comparison of TMA cores with whole slide evaluation, strongly correlated when assuming a linear monotonic relation. Potential explanations for the significant discordances using the Wilcoxon ranked sign test could be (1) the pronounced discordance in cases with PD-L1 TPS greater than or equal to 10% (Supplementary Figs. 5–7), in which high variability is inherent to human evaluation, and (2) the smaller sample size compared with the BM tissue cohort (n = 84 versus n = 191 for BM). Nevertheless, as TMA cores are mirroring small tumor biopsies and discordance was present, caution must be taken when assessing PD-L1 on biopsy samples from the primary lung tumor, especially should future studies introduce more subtle cutoffs. As a result, it has been recommended to take at least four biopsy samples to reach high concordance with whole tissue sections.^35^

However, we found no intralesional heterogeneity in the BM samples and there was no statistically significant heterogeneity when comparing whole slides with TMA cores, increasing confidence in PD-L1 assessment on small stereotactic biopsies. This discrepancy of heterogeneity between the primary tumor and BM may be biologically explained by the multistep invasion cascade.^30^ In a heterogeneous primary tumor with multiple clones, one clone detaches and disseminates systemically until it forms a metastasis.^30^ Brastianos et al.^36^ revealed that intracranial metastases were genetically homogeneous, including potentially clinically informative driver alterations, although genetically divergent from their primary tumor. This finding could also apply to PD-L1 expression.

The prognostic value of PD-L1 TPS in advanced NSCLC has been investigated in several studies with discrepant results, depending on the used antibody clone, staining protocols, and cutoff values.^28^^,^^37^^,^^38^ In our cohort of 191 patients with BM, PD-L1 expression had no significant prognostic value regarding OS or BMOS.

In conclusion, we found that PD-L1 scores are concordant between most paired primary lung carcinomas and their BM. Discordant cases could not be explained by longer time periods between tissue sampling or administration of chemotherapy or radiotherapy between tissue acquisition, nor by limited tissue availability from the primary tumor, for example, owing to biopsies only, and may be owing to tumor heterogeneity in the primary tumor and metastasis of selected clones, resulting in increased homogeneity of the BM.

## CRediT Authorship Contribution Statement

**Alexandra Kündig:** Investigation, Data curation, Writing—original draft, Writing—review and editing.

**Philipp Zens:** Software, Formal analysis, Data curation, Visualization, Funding acquisition, Writing—original draft, Writing—review and editing.

**Christian Fung, Amina Scherz, Ferdinando Cerciello:** Investigation, Resources, Writing—review and editing.

**Evelyn Herrmann, Ralph A. Schmid:** Resources, Writing—review and editing.

**Ekin Ermis:** Investigation, Writing—review and editing.

**Erik Vassella:** Resources, Data curation, Writing—review and editing.

**Sabina Berezowska:** Conceptualization, Methodology, Resources, Data curation, Writing—original draft, Writing—review and editing, Visualization, Supervision, Project administration, Funding acquisition.
