# Supplementary material for: Programmed Death-Ligand 1 Expression in Lung Cancer and Paired Brain Metastases—a Single-Center Study in 190 Patients
Source: JTO Clin Res Rep. 2022 Sep 20;3(11):100413. doi: 10.1016/j.jtocrr.2022.100413 (PMC9579806; doi:10.1016/j.jtocrr.2022.100413)
Supplement: Supplementary Material [file mmc1.docx]

# **Supplementary material**

| Supplementary Figure 1 | Flow Chart of patients in the study | Page 1 |
| --- | --- | --- |
| Supplementary Figure 2 | PD-L1 TPS is comparable between samples of different age | Page 2 |
| Supplementary Figure 3 | Bland-Altman plot comparing PD-L1 TPS assessment of primary lung carcinoma and the paired brain metastasis | Page 3 |
| Supplementary Figure 4 | Higher age is associated with discordant PD-L1 expression (primary lung carcinoma and brain metastasis) regarding the 1% cut-off | Page 4 |
| Supplementary Figure 5 | Comparison of PD-L1 TPS assessment between whole slides and TMA | Page 5 |
| Supplementary Figure 6 | Intercore and interregion concordance of PD-L1 TPS assessed in the TMA for brain metastases | Page 6 |
| Supplementary Figure 7 | Intercore and interregion concordance of PD-L1 TPS assessed in the TMA for primary lung carcinoma | Page 7 |
| Supplementary Figure 8 | Lack of prognostic relevance of concordance status for PD-L1 TPS between primary lung tumor and brain metastasis | Page 8 |
| Supplementary Table 1 | Major genomic alterations present in the subset of patients where NGS analysis was performed | Page 9 |
| Supplementary Table 2 | Cases with multiple BM or multiple slides of the same BM assessed for PD-L1 | Page 10 |
| Supplementary Table 3 | Association of mutational subgroup with discordant PD-L1 expression between the primary lung carcinoma and brain metastasis | Page 11 |

## **Supplementary figure 1:** Flow Chart of patients in the study

Suppl. Figure 1: Flow chart of patients in the study. Of the initial 212 patients, 191 were included for PD-L1 assessment using whole slides. For 84/191 patients, PD-L1 assessment was also possible in paired primary lung carcinoma. For 187/191 patients, PD-L1 could also be assessed in TMA cores of the tumor. In 16/191 cases PD-L1 was assessed on multiple brain metastases or multiple slides from the same brain metastasis.

## **Supplementary figure 2:** PD-L1 TPS is comparable between samples of different age

##

Suppl. Figure 2: PD-L1 TPS was comparable between samples (= paraffin blocs) of different age. There was no significant overall difference of PD-L1 TPS between samples processed and diagnosed over a time span of 16 years (p = 0.744).

**Supplementary figure 3:** Bland-Altman plot comparing PD-L1 TPS assessment of primary lung carcinoma and the paired brain metastasis

**
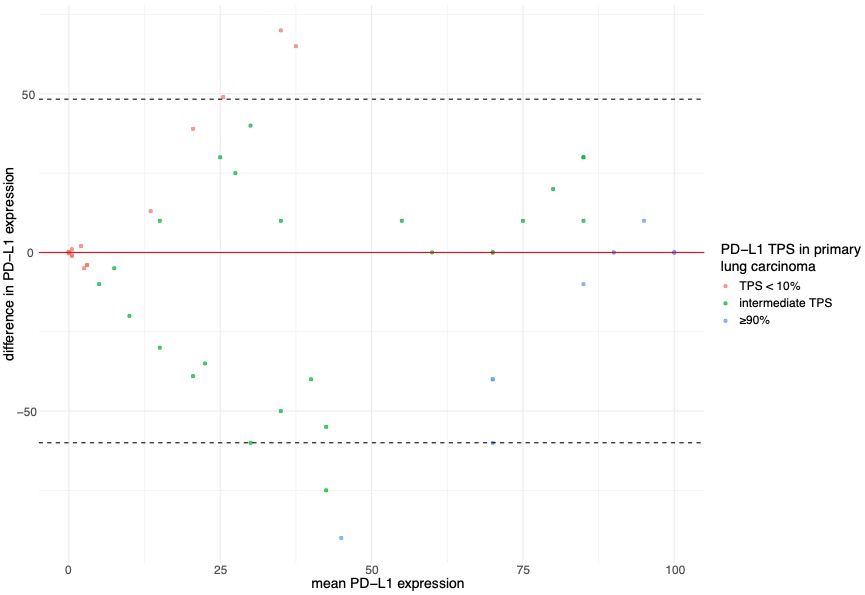
**

Suppl. Figure 3: Bland-Altman plot comparing PD-L1 TPS assessment of primary lung carcinoma and the paired brain metastasis. There was no significant difference when comparing the crude PD-L1 TPS assessed in the primary lung carcinoma or paired brain metastases (p = 0.567). However, especially for cases with TPS PD-L1 ≥10% in the primary lung carcinoma there is a certain variability between the two sites.

# **Supplementary figure 4:** Higher age is associated with discordant PD-L1 expression (primary lung carcinoma and brain metastasis) regarding the 1% cut-off

Suppl. Figure 7: Boxplot visualizing that higher patient age is significantly associated with discordant PD-L1 TPS regarding the 1% cut-off when comparing BM and paired primary lung carcinoma.

## **Supplementary figure 5:** Comparison of PD-L1 TPS assessment between whole slides and TMA


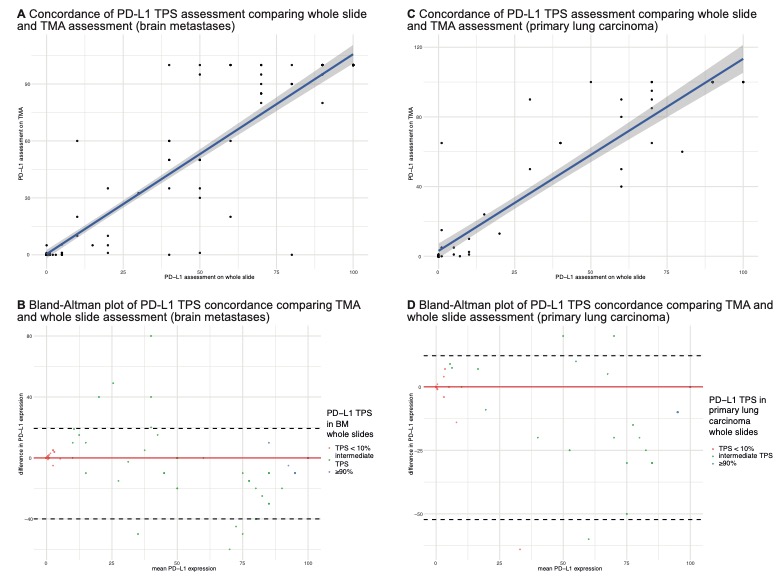


Suppl. Figure 4: PD-L1 tumor proportional scores assessed in the whole slides or the TMA cores are concordant in the brain metastases (p_Wilcoxon signed-rank_ = 0.05, p_Spearman_ < 0.001, r_Spearman_ = 0.91; A, B) but less concordant in the primary lung carcinoma (p_Wilcoxon signed-rank_ = 0.001, p_Spearman_ < 0.001, r_Spearman_ = 0.94; C, D).

# **Supplementary figure 6:** Intercore and interregion concordance of PD-L1 TPS assessed in the TMA for brain metastases


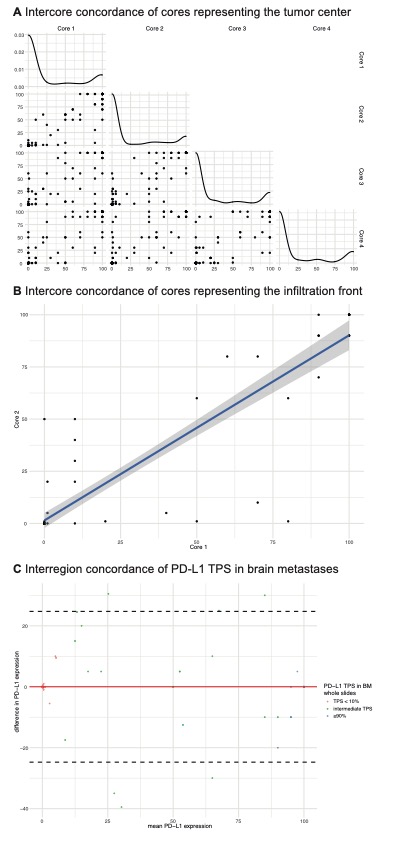


Suppl. Figure 5: Concordance of PD-L1 TPS assessed in brain metastases between TMA cores from the tumor center (A), the infiltration front (B) and between the different regions (C). Colors are depicting the TPS as assessed in the corresponding whole slides.

# **Supplementary figure 7:** Intercore and interregion concordance of PD-L1 TPS assessed in the TMA for primary lung carcinoma

##
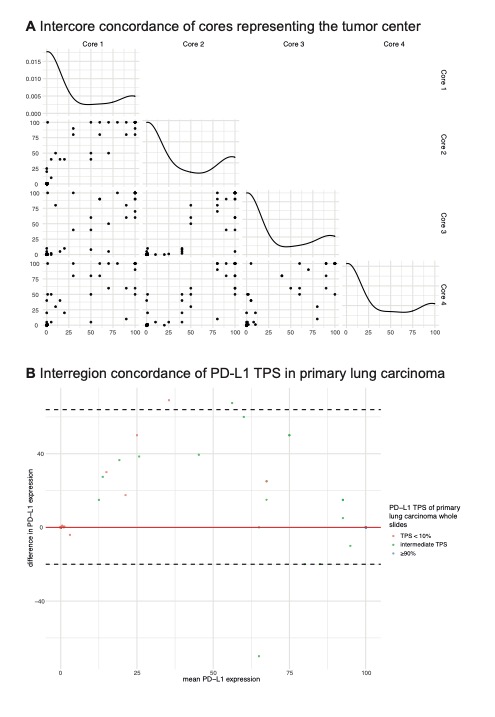


Suppl. Figure 6: Concordance of PD-L1 TPS assessed in primary lung carcinoma between TMA cores from the tumor center (A) and between the different regions (B). Colors are depicting the TPS as assessed in the corresponding whole slides.

# **Supplementary figure 8:** Lack of prognostic relevance of concordance status for PD-L1 TPS between primary lung tumor and brain metastasis

Suppl. Figure 8: Survival plots depicting the lack of prognostic significance of PD-L1 TPS for overall survival (A) and brain specific overall survival (B).

# **Supplementary table 1:** Major genomic alterations present in the subset of patients where NGS analysis was performed

|  | **None** | **P = BM** | **P < BM** | **P > BM** | **P / BM** |
| --- | --- | --- | --- | --- | --- |
| **EGFR** | 49 | 4 |  |  |  |
| **ALK** | 52 | 1 |  |  |  |
| **ROS1** | 53 |  |  |  |  |
| **BRAF** | 53 |  |  |  |  |
| **NTRK 1/2** | 53 |  |  |  |  |
| **MET** | 47 | 4 | 2 |  |  |
| **ERBB2** | 52 | 1 |  |  |  |
| **STK11** | 46 | 7 |  |  |  |
| **KRAS** | 18 | 28 | 3 | 3 | 1 |

Suppl. Table 1: Driver mutations and genetic alterations with potential impact on PD-L1 expression. P = BM, same alterations in the primary lung carcinoma and brain metastasis; P < BM, additional alterations in the brain metastasis; P > BM additional alterations in the primary lung carcinoma; P / BM, different alterations in primary lung carcinoma compared to the brain metastasis; P, primary lung carcinoma; BM, brain metastasis

# **Supplementary table 2:** Cases with multiple BM or multiple slides of the same BM assessed for PD-L1

| **PID** | **TPS primary lung carcinoma [%]** | **TPS BM1 [%]** | **TPS BM2 [%]** | **TPS BM3 [%]** | **comments** |
| --- | --- | --- | --- | --- | --- |
| 23 |  | 0 | 1 |  | 2 different BM at 1 month interval |
| 27 |  | 50 | 20 |  | 2 different BM at ½ month interval |
| 46 |  | 0 | 0 | 0 | 2 different BM at 7 months interval + 2 slides of 2^nd^ BM |
| 64 | 90 | 100 | 90 | 90 | 3 different BM at 4 respectively 10 months interval |
| 93 | 10 | 40 | 50 |  | 2 slides from same BM |
| 103 |  | 0 | 0 |  | 2 slides from same BM |
| 109 | 0 | 0 | 0 |  | 2 different BM at 3 months interval |
| 117 | 1 | 3 | 5 |  | 2 different BM at 5 months interval |
| 132 |  | 0 | 0 |  | 2 different BM at 12 months interval |
| 138 |  | 50 | 60 |  | 2 different BM at 7 days interval |
| 144 | 0 | 0 | 0 |  | 2 different BM at 6 months interval |
| 159 | 0 | 0 | 0 |  | 2 different BM at 14 months interval |
| 176 |  | 0 | 0 |  | 2 different BM at 14 months interval |
| 177 |  | 0 | 1 |  | 2 different BM at 32 months interval |
| 179 | 0 | 0 | 0 |  | 2 different BM at 25 months interval |
| 197 | 90 | 90 | 90 |  | 2 different BM at 10 months interval |

Suppl. Table 2: Cases for which tissue from multiple brain metastases or multiple slides was assessed for PD-L1 expression. PID, patient identificator; TPS, tumor proportional score; BM, brain metastasis

# **Supplementary table 3:** Association of mutational subgroup with discordant PD-L1 expression between the primary lung carcinoma and brain metastasis

|  | **Concordant**  **(50% cut-off)** | **Discordant**  **(50% cut-off)** | **Total** |
| --- | --- | --- | --- |
| **P = BM (%)** | 9 (90) | 1 (10) | 10 |
| **P > BM (%)** | 11 (91.7) | 1 (8.3) | 12 |
| **P < BM (%)** | 12 (92.3) | 1 (7.7) | 13 |
| **P ∩ BM (%)** | 12 (92.3) | 1 (7.7) | 13 |
| **P / BM (%)** |  | 4 (100) | 4 |
| **Total** | 44 (84.6) | 8 (15.4) | 52 |

Suppl. Table 3: Patient with no shared genomic alteration between the primary lung carcinoma and brain metastasis are all discordant regarding the 50% cut-off
